# Changing the Game: Using Integrative Genomics to Probe Virulence Mechanisms of the Stem Rust Pathogen *Puccinia graminis* f. sp. *tritici*

**DOI:** 10.3389/fpls.2016.00205

**Published:** 2016-02-24

**Authors:** Melania Figueroa, Narayana M. Upadhyaya, Jana Sperschneider, Robert F. Park, Les J. Szabo, Brian Steffenson, Jeff G. Ellis, Peter N. Dodds

**Affiliations:** ^1^Department of Plant Pathology and the Stakman-Borlaug Center for Sustainable Plant Health, University of MinnesotaSt. Paul, MN, USA; ^2^Agriculture, Commonwealth Scientific and Industrial Research OrganisationCanberra, ACT, Australia; ^3^Agriculture, Centre for Environment and Life Sciences, Commonwealth Scientific and Industrial Research OrganisationPerth, WA, Australia; ^4^Faculty of Agriculture and Environment, Plant Breeding Institute, The University of SydneyNarellan, NSW, Australia; ^5^Cereal Disease Laboratory, United States Department of Agriculture-Agricultural Research ServiceSt. Paul, MN, USA

**Keywords:** *Puccinia*, effectors, resistance, stem rust, virulence, avirulence, wheat, barley

## Abstract

The recent resurgence of wheat stem rust caused by new virulent races of *Puccinia graminis* f. sp. *tritici* (*Pgt*) poses a threat to food security. These concerns have catalyzed an extensive global effort toward controlling this disease. Substantial research and breeding programs target the identification and introduction of new stem rust resistance (*Sr*) genes in cultivars for genetic protection against the disease. Such resistance genes typically encode immune receptor proteins that recognize specific components of the pathogen, known as avirulence (Avr) proteins. A significant drawback to deploying cultivars with single *Sr* genes is that they are often overcome by evolution of the pathogen to escape recognition through alterations in *Avr* genes. Thus, a key element in achieving durable rust control is the deployment of multiple effective *Sr* genes in combination, either through conventional breeding or transgenic approaches, to minimize the risk of resistance breakdown. In this situation, evolution of pathogen virulence would require changes in multiple *Avr* genes in order to bypass recognition. However, choosing the optimal *Sr* gene combinations to deploy is a challenge that requires detailed knowledge of the pathogen *Avr* genes with which they interact and the virulence phenotypes of *Pgt* existing in nature. Identifying specific *Avr* genes from *Pgt* will provide screening tools to enhance pathogen virulence monitoring, assess heterozygosity and propensity for mutation in pathogen populations, and confirm individual *Sr* gene functions in crop varieties carrying multiple effective resistance genes. Toward this goal, much progress has been made in assembling a high quality reference genome sequence for *Pgt*, as well as a Pan-genome encompassing variation between multiple field isolates with diverse virulence spectra. In turn this has allowed prediction of *Pgt* effector gene candidates based on known features of *Avr* genes in other plant pathogens, including the related flax rust fungus. Upregulation of gene expression in haustoria and evidence for diversifying selection are two useful parameters to identify candidate *Avr* genes. Recently, we have also applied machine learning approaches to agnostically predict candidate effectors. Here, we review progress in stem rust pathogenomics and approaches currently underway to identify *Avr* genes recognized by wheat *Sr* genes.

## Introduction

The basidiomycete fungus *Puccinia graminis* f. sp. *tritici* (*Pgt*) causes stem rust or black rust, one of the most devastating diseases affecting common and durum wheat, barley, and triticale (Leonard and Szabo, 2005; Park, 2007). While both barley and triticale are valuable dietary and industrial crops, the menace that stem rust poses to wheat production is the most feared. Wheat provides one fifth of the calories and protein intake for human consumption across the globe. The world's wheat production for 2015 is estimated to be more than 733 million tons (FAO, 2015), a number that will need to be more than doubled to meet supply demands projected by the year 2050 (Wheat, 2014). *Pgt* has a wide geographical distribution, and it can destroy a wheat field entirely in as little as 3 weeks (Leonard and Szabo, 2005). For these reasons, *Pgt* has been ranked as one of the most economically important plant pathogens and threats to global food security (Dean et al., 2012).

In 1998, a new highly virulent race of *Pgt*, referred to as TTKSK (synonym Ug99), was detected in Uganda (Pretorius et al., 2000). This race overcame the long term protection that had been provided by the stem rust resistance gene *Sr31*, used widely in many parts of the world. Since then, at least eight variants of the Ug99 race group have appeared, rendering additional resistance genes ineffective (Singh et al., 2011, 2015). The steady geographical spread and rapid evolution of the Ug99 race group poses a significant threat, with 80 and 95% of global wheat and barley cultivars respectively considered susceptible (Singh et al., 2011; Steffenson et al., 2013). This threat has led to a major worldwide investment in improving genetic resistance to wheat stem rust through identifying and incorporating new sources of resistance effective against this race group. However, in 2013-2014, Ethiopia suffered a devastating stem rust outbreak caused by an unrelated *Pgt* race (TKTTF) which defeated the resistance gene *SrTmp* in the widely cultivated wheat variety “Digalu,” introduced to provide protection against the Ug99 race group (Olivera et al., 2015). Similarly, in 2014 Kenya experienced significant losses in fields of the wheat variety “Robin,” which also carries *SrTmp*, in this case caused by a new race in the Ug99 lineage (Singh et al., 2015; Patpour et al., 2016). Thus, there is an ongoing need to not only diversify sources of resistance with a broader focus beyond the Ug99 lineage, but also to ensure that multiple *Sr* genes are deployed in combination to guard against future stem rust epidemics.

Currently the best strategy for achieving long-lasting resistance involves the use of several different stem rust resistance (*Sr*) genes with complementary race specificity, in combination with non-specific partial resistance genes (Ellis et al., 2014). However, there is a challenge in identifying the optimal genes and gene combinations to minimize the chance of new virulent races arising. Because most *Sr* genes confer race-specific resistance based on recognition of pathogen avirulence (*Avr*) genes (McIntosh et al., 1995; Zambino et al., 2000), knowledge of the specific genes underlying virulence and their population genetics is critical to informing resistance deployment strategies. What are the pathogen *Avr* genes recognized by important *Sr* genes? How variable are they in local and global *Pgt* populations? What is the level of homozygosity/heterozygosity at these loci and thus their propensity to mutate to virulence? Some *Sr* genes from different sources cannot be distinguished because they provide resistance to all known pathotypes of *Pgt*, but do they in fact recognize different *Avr* genes? Lastly, the recent cloning of several *Sr* genes, including *Sr33* (Periyannan et al., 2013), *Sr35* (Saintenac et al., 2013), *Sr50* (Mago et al., 2015), *Sr55* (Moore et al., 2015), and *Sr57* (Krattinger et al., 2009) brings the possibility of developing multigene transgenic stacks that segregate as a single locus (Ellis et al., 2007; Wulff et al., 2011; McDonald, 2014). Because pyramids of multiple broadly effective resistance genes confound the confirmation of individual gene function through pathogenicity assays, it will be important to generate tools allowing their independent assay based on knowledge of their corresponding *Avr* genes. Here, we discuss the implications of recent advances in *Pgt* molecular genetics and genomics in the context of these research priorities and the underlying biology and history of stem rust disease.

## Biology of the stem rust fungus: Mastering complexity to survive

**Figure 1:**
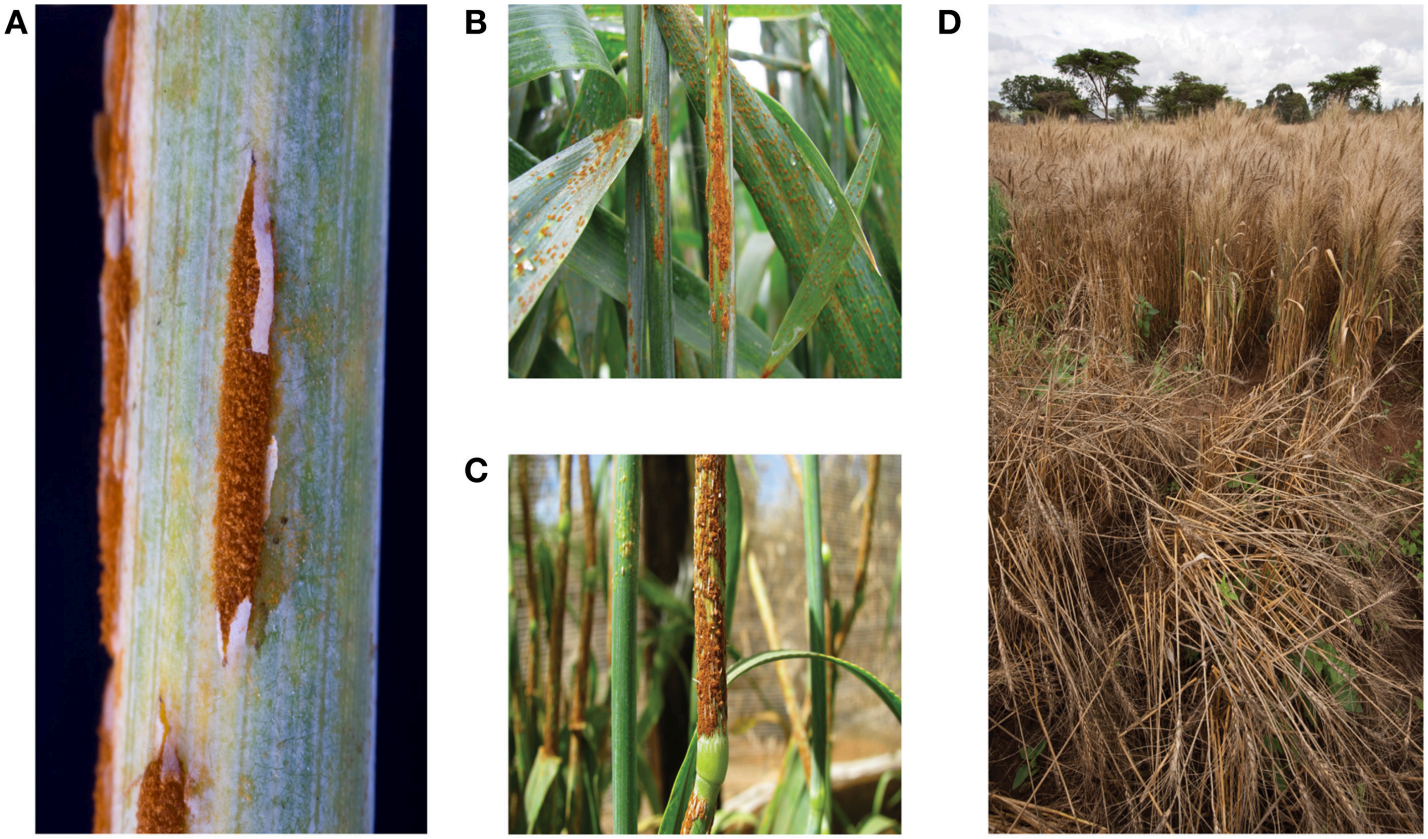
*****P. graminis*** f. sp. ***tritici*** (***Pgt***) infected wheat and barley. (A)** Close-up of a *Pgt* uredinium after eruption through the epidermis of a barley stem. **(B)** Sporulation of African *Pgt* race PTKST on wheat **(C)** Sporulation of African *Pgt* race PTKST on barley. **(D)** Severe straw breakage of a susceptible wheat line (front) caused by heavy stem rust infection in comparison with a resistant wheat line (back) growing in a disease screening nursery in Kenya in 2008. Images **(A,D)** were taken by David Hansen/Brian Steffenson and **(B,C)** by Zacharias Pretorius.

*Pgt* is a biotrophic fungus with a complex life cycle that includes asexual (clonal) reproduction on a cereal host (Poaceae), such as wheat, and sexual recombination on an alternate host (Berberidaceae; Leonard and Szabo, 2005). During the asexual phase, the fungus produces multitudes of single-celled dikaryotic urediniospores, which can mediate new cycles of infection approximately every 2 weeks. Urediniospores germinate on the leaf surface after exposure to moisture (Staples and Macko, 2004; Leonard and Szabo, 2005). The topology of the leaf surface helps the germ tube to locate a stomate where a specialized penetration structure, known as an appressorium, forms. The appressorium remains quiescent over the stomatal aperture until the formation of a penetration peg is triggered by exposure of the plant to light (Yirgou and Caldwell, 1968). Subsequently, the fungus forms a substomatal vesicle that gives rise to the primary infection hypha (Rowell et al., 1958). As the mycelium grows and comes into contact with a mesophyll cell, the fungus produces a specialized feeding structure, known as a haustorium (Harder and Chong, 1984; Staples and Macko, 2004). After formation of the first haustorium, the fungus branches to generate secondary infection hyphae and form additional haustoria. The haustorium penetrates the plant cell wall, and invaginates the plasma membrane creating a sealed compartment, referred to as the extrahaustorial matrix (Harder and Chong, 1984). In addition to nutrient uptake, these structures enable the delivery of effector proteins into the host cell that are thought to manipulate cellular processes to facilitate infection (Garnica et al., 2014). These elaborate morphological and molecular events take place during the first days of *Pgt* infection and macroscopic symptoms of disease usually manifest after 8–10 days, as urediniospore pustules erupt through the leaf or stem surface (Figures 1A–C; Leonard and Szabo, 2005). Yield reduction associated with stem rust infection in cereals is attributed to plant lodging, poor nutrient mobilization to support grain development, and decrease of photosynthetic capacity as the plant foliar area is severely damaged (Figure 1D; Leonard and Szabo, 2005).

During the asexual stage described above *Pgt* is dikaryotic (n + n), with each cell containing two haploid nuclei **dikaryotic genetics**. However, toward the end of the growing season, as the cereal host starts to senesce, the fungus may initiate the sexual stage through formation of diploid (2*n*) teliospores. These thick-walled, two-celled spores can remain dormant until the spring and then complete meiosis to generate haploid basidiospores that can infect the alternate host *Berberis* (barberry) or *Mahonia* (Roelfs, 1985; Roelfs and Groth, 1988; Boehm et al., 1992). The haploid infection stage culminates in the production pycnia, which contain two types of haploid gametes (Roelfs, 1985). Haploid pycniospores cross fertilize with receptive hyphae to re-establish dikaryotic mycelia that give rise to aeciospores, which can then infect the cereal host and re-initiate the asexual phase (Harder, 1984). Thus, in areas where the alternate host exists, the sexual cycle of the fungus can yield novel virulence phenotypes due to re-assortment and segregation of genetic variation.

KEY CONCEPT 1 **Dikaryotic genetics**

*Pgt* is a dikaryotic pathogen with an asexual cycle on wheat and barley, and a sexual cycle on an alternate host. *Pgt* therefore exhibits diploid genetics, with a virulent phenotype requiring homozygosity at the *Avr* locus.

## Genetic resistance to stem rust disease: Racing evolution

Race-specific resistance (*R*) genes, often known as “major” or “seedling” resistance genes in the wheat and barley context, are one of the fundamental resources utilized in disease resistance breeding programs. These *R* genes generally conform to the “gene-for-gene” model, and confer resistance to pathogen races carrying a corresponding *Avr* gene (Flor, 1971) **race-specific resistance**. Most known *R* genes encode intracellular immune receptor proteins belonging to the nucleotide-binding leucine-rich-repeat (NB-LRR) class (Dangl and Jones, 2001; Dodds and Rathjen, 2010). These receptors can recognize specific pathogen effector proteins (known as Avr proteins) that are translocated into the plant cell (Hogenhout et al., 2009; Koeck et al., 2011).

KEY CONCEPT 2 **Race-specific resistance**

Race-specific resistance to *Pgt* is mediated by ‘gene-for-gene’ type interactions that involve recognition of pathogen avirulence proteins by host resistance proteins.

The flax rust disease system provides the best defined model for rust disease resistance, with over 30 corresponding *R* and *Avr* genes defined genetically in the host (*Linum usitatissimum*) and pathogen (*Melampsora lini*), respectively, many of which are now cloned (Ravensdale et al., 2011). In this system, all the known *R* genes encode NB-LRR intracellular receptors, while the corresponding *Avr* genes encode small secreted proteins that are expressed in haustoria and delivered into host cells. There are over 50 race-specific stem rust resistance (*Sr*) genes described in wheat and its close relatives (McIntosh et al., 1995), a number of which have been cloned recently (Periyannan et al., 2013; Saintenac et al., 2013; Mago et al., 2015). As in flax, these all encode NB-LRR immune receptors, suggesting that the corresponding *Pgt* Avr proteins are likely to be secreted effectors delivered to host cells. Likewise in barley, the *Rpg4/5* stem rust resistance locus encodes a pair of NB-LRR receptors that probably function in tandem (Brueggeman et al., 2008; Wang et al., 2013). Although *R* genes encoding cell surface immune receptors that recognize apoplastic effectors have been identified in some disease systems (Tax and Kemmerling, 2012; Du et al., 2015), such receptors have not been observed in rust resistance. However, the barley *Rpg1* resistance gene is unusual in encoding an intracellular kinase (Brueggeman et al., 2002) and appears to respond to components present in the urediniospore that may be released early in infection (Nirmala et al., 2011).

*Avr* genes in flax rust are characterized by high levels of polymorphism between races and display signatures of diversifying selection (Catanzariti et al., 2006; Dodds et al., 2006; Barrett et al., 2009) as a result of the strong pressure to escape host immune recognition **avirulence proteins**. For instance, 12 variants of AvrL567 are known with different recognition properties determined by polymorphic residues exposed on the protein surface that interact with host immune receptors (Wang et al., 2007; Moore et al., 2015). Likewise, differences in recognition of AvrM variants by the corresponding M resistance protein are governed by surface-exposed polymorphic residues (Catanzariti et al., 2010; Ve et al., 2013). Effectors are probably also under selection to adapt to host proteins targeted by their pathogenicity functions. The only other basidiomycete avirulence gene identified to date is *UhAvr1* from *Ustilago hordei* (causal agent of barley smut) and in this case virulence arose from a transposable element insertion in the promoter of the gene (Ali et al., 2014).

KEY CONCEPT 3 **Avirulence proteins**

Known Avr proteins of rust fungi are secreted from haustoria and delivered into host cells where they are recognized by NB-LRR immune receptors. Thus, there is strong selection pressure for *Avr* sequence diversification to allow the pathogen to escape recognition.

Additional genetic protection against rust pathogens can also occur through the action of race non-specific resistance genes. In wheat, some of these genes provide broad-spectrum but partial resistance against multiple pathogens and probably operate by physiological mechanisms independently of immune recognition. For instance *Lr34* (syn. *Sr57, Yr18, Pm38*) confers resistance against stem, leaf and stripe, rust fungi as well as barley yellow dwarf virus and powdery mildew and encodes a putative adenosine triphosphate-binding cassette (ABC) transporter protein (Dyck et al., 1966; Singh, 1993; Krattinger et al., 2009; Risk et al., 2013). Likewise, *Lr67* (syn. *Sr55, Yr46, Pm46*) also confers multi-pathogen resistance and encodes a hexose transporter (Moore et al., 2015). Because these non-specific genes may also enhance the effectiveness of race-specific genes, a desirable strategy to enhance genetic resistance is to employ combinations of both resistance gene classes (Ellis et al., 2014). Indeed North America, Mexico and Australia have been largely protected from stem rust epidemics since the 1950s, probably due to the continuous deployment of rust resistant wheat cultivars often with multiple resistance genes in the absence of significant sexual recombination in the pathogen populations (Leonard and Szabo, 2005; Park, 2007; Ellis et al., 2014).

## Genetic variability of *Pgt*: The code for success

A significant challenge for resistance breeding is the “boom and bust” cycle resulting from the emergence of new pathogen variants or races that overcome single major resistance genes. Although many *Sr* genes have been deployed in the field, resistance breakdown occurs frequently due to genetic changes in existing local *Pgt* races or the migration of new races, as exemplified by the Ug99 race group (Stokstad, 2007) **sources of genetic variation**. Indeed, *Pgt* is genetically diverse and many different physiological races have been described by systematic classification systems based on the disease phenotypes (infection types) induced in a standard set of differential lines containing known *Sr* genes (Roelfs and Martens, 1988; Jin et al., 2008). The differential host set has often been modified or extended in different regions based on locally important *Sr* genotypes (Park, 2008). These classification systems reflect the genetic make-up of the *Avr* gene repertoires within isolates, and have been a very useful tool to monitor pathogen types in field surveys and detect changes in virulence.

KEY CONCEPT 4 **Sources of genetic variation**

Genetic variability in *Pgt* can occur via multiple mechanisms, such as random mutations in clonal lineages, sexual recombination in geographical regions where the alternate host is present, and somatic hybridization.

Stem rust epidemics occur intermittently in regions with warm and humid weather conditions and references to recurrent disease outbreaks date back to ancient Roman times (Dubin and Brennan, 2009). Stem rust epidemics have impacted wheat production in the United States (US) from the late 1800's. The two most devastating epidemics occurred in 1935 and 1953-1954. In the epidemic of 1935 yield losses recorded in the upper Midwest (Minnesota and North Dakota) reached more than 50% (Roelfs, 1978). The 1953-1954 epidemics were responsible for the loss of more than 75% of the US durum wheat (Stakman and Harrar, 1957). In response to disease outbreaks in the early 1900s, a barberry eradication program was initiated throughout the northern Great Plains and lasted for nearly a half of century. As a result the number of epidemics and diversity of the *Pgt* population has been dramatically reduced (Roelfs, 1982). Since then, there has been only one epidemic reported to the US and Canada in 1974 (Roelfs, 1978; Leonard and Szabo, 2005). Currently, in the primary wheat growing regions of the United States (east of the Rocky Mountains) the *Pgt* population is primarily composed of a single race and stem rust is rarely observed in commercial wheat production fields (Jin et al., 2014). Another important factor that has helped reduce the complexity of the *Pgt* population in Great Plains is the inability of the stem rust pathogen to survive the winter season in the colder regions, except for along the Gulf Coast. Due to this genetic bottleneck, the development of stem rust in the central and northern Great Plains is dependent on the transport of fresh inoculum from the Gulf Coast region. This annual northward movement of fungal spores has been referred to as the Puccinia Pathway. The use of rust resistant varieties in Mexico by CIMMYT in the 1950's and 1960's, led by Norman Borlaug (Borlaug, 1968) may have decreased the amount of inoculum migrating from Northern Mexico into the Puccinia Pathway, and thus helped to reducing the severity and incidence of the disease.

Similarly, Australia has also struggled with the economic losses caused by *Pgt*. Some of the significant epidemics of stem rust in Australia occurred in the late 1800's, followed by the 1947-1948 epidemic when the state of New South Wales lost ~12% of grain yield (Stakman and Harrar, 1957; Dubin and Brennan, 2009) and finally in 1973 when the country faced the worst outbreak leading to AU$100-200 million losses (Watson, 1981; McIntosh et al., 1995; Park, 2007). Surveys of *Pgt* have indicated that it was introduced into the Australian continent on at least three occasions since 1921 (Park, 2007). Because barberry is not present in Australia, the pathogen has evolved largely clonally with three or four independent lineages recognized, each derived from an independent introduction. The annual surveys of *Pgt* races across the Australian wheat belt for over 90 years provide a detailed history of changes in virulence in these lineages over time (Park, 2007). The predominant evolutionary mechanism involves changes in which single virulence phenotypes are acquired sequentially by new races within a *Pgt* lineage. This is most likely due to mutations occurring within single *Avr* genes. For example, the detection of virulence for the resistant wheat cultivars Eureka (*Sr6*), Gabo (*Sr11*), Spica (*Sr17*), and Festival (*Sr9b*) soon after their release was considered to be the result of mutation (Waterhouse, 1952; Watson, 1958). Experiments under controlled conditions demonstrated the acquisition of virulence for resistance genes following exposure to the chemical mutagen Ethyl Methane Sulphonate (Luig, 1978; Gates and Loegering, 1991). Luig (1978) showed that *Avr* genes vary in their mutability, with mutations to virulence for the resistance genes *Sr5, Sr9e*, and *Sr21* being relatively common, while mutations to virulence for *Sr26* were not observed. It was suggested that the *Avr* genes present in *Pgt* have intrinsic differences. Because *Pgt* is dikaryotic, the probability of virulence arising should differ according to whether a single mutation (in a race heterozygous for avirulence) or two mutations (in a homozygote) are required. This may explain some of the observed differences in the durability of *Sr* genes in particular environments with varying pathogen populations. Alternatively, some virulence mutations may impose fitness costs.

Somatic hybridization, which involves fusion of dikaryotic hyphae from different isolates and either nuclear exchange or parasexual recombination and exchange of chromosomes, is another mechanisms that may introduce variability in the absence of sexual recombination (Park and Wellings, 2012). For instance, the Australian pathotype Pgt 34-2-11 probably emerged as the result of somatic hybridization between isolates belonging to race 126 and race 21 (Watson, 1981). Wang and McCallum found that germinating urediniospores of the wheat leaf fungus, *Puccinia triticina*, can undergo anastomosis and nuclear migration (Wang and McCallum, 2009). The genomics tools now available will help to determine the frequency and role of somatic hybridization in the evolution of *Pgt* populations.

Clearly, identifying *Avr* genes from *Pgt* is a priority to enhance crop protection strategies. However, the biotrophic lifestyle of rust fungi has posed experimental constraints on development of genetic tools, such as transformation and gene-knockout strategies, that are used in other fungi. Sequencing advances have greatly enhanced the amenability of these pathogens for experimental analysis, with genome assemblies now available for several rust fungi, including those causing wheat stem, leaf and stripe rust, poplar leaf rust, and flax rust (Duplessis et al., 2011; Cantu et al., 2013; Zheng et al., 2013; Nemri et al., 2014) (http://www.broadinstitute.org/annotation/genome/puccinia_group/MultiHome.html).

A high quality reference genome sequence for a North American *Pgt* isolate, CDL 75-36-700-3, was assembled based on Sanger sequencing of plasmid and fosmid clones (Duplessis et al., 2011). This approach resulted in a highly connected assembly spanning 88.6 Mbp in just 392 scaffolds of 4557 contigs (81.5 Mbp sequence length). A total of 15,979 genes were annotated based on a combination of *in silico* predictions, EST sequences and RNAseq transcript analysis. Resequencing of the *Pgt* reference isolate using Illumina 76b paired-end reads identified about 129,000 single nucleotide polymorphisms (SNPs) between the two haploid genomes in this isolate. Subsequent analysis of global isolates has led to the development of a high throughput SNP genotyping array. The PgtSNP 1.5 k chip has been used to genotype *Pgt* isolates from Northeast Africa to examine the genetic relationship between races of *Pgt* (Olivera et al., 2015). This analysis clearly demonstrated that the *Pgt* race TKTTF, which caused the 2013-14 wheat stem rust epidemic in Ethiopia, was not derived from Ug99 lineage or other common races in the region. In addition, the Ethiopian isolates of race TKTTF were found to represent two distinct subclades, indicating that evolution of this race was not a recent event. Current genotyping of *Pgt* isolates from Europe, Middle East and Central Asia supports this hypothesis. Thus, SNP genotyping provides a rapid tool for surveillance of critical *Pgt* race groups in regions where populations are primarily asexual. In addition, this tool allows population genetics studies to examine *Pgt* population structure and evolution and is currently being use to analyze the Ug99 lineage. An important question to address is whether sexual recombination contributes to *Pgt* population diversity in Africa.

The reference sequence has also provided an excellent resource to facilitate broader genomic analyses of *Pgt*. Upadhyaya et al. (2015) used this reference in combination with Illumina sequencing to build a pan-genome of Australian *Pgt* isolates. This was based on sequences of isolates representing founder races for the four clonal lineages present in Australia. In addition to the reference-based assembly, a *de novo* assembly of unaligned reads was also used to give a final 95 Mbp pan-genome. The additional ~15 Mbp *de novo* assembled sequence in this genome is not race specific, because it was present in all four independent Australian *Pgt* isolates, and indeed also in unassembled reads from CDL 75-36-700-3. Only low amounts of race-specific sequence, < 0.5 Mbp, represented by numerous short contigs, were observed. This suggests that there are few large scale genome structural differences between *Pgt* races, unlike observations in other pathogenic fungi such as *Fusarium* (Ma et al., 2010) and *Magnaporthe* (Yoshida et al., 2009). However, very high rates of heterozygosity were observed, with around a million SNPs detected within each race representing diversity between the two haploid nuclei (Upadhyaya et al., 2015).

Identifying putative *Avr* genes in the *Pgt* genome is not simple. The key criteria that can be extrapolated from the known flax rust *Avr* genes are quite broad; they should encode secreted proteins and should be expressed in haustoria **effector candidates**. In contrast to oomycetes (Bozkurt et al., 2012), there are no conserved sequence motifs uniting these genes into a readily identified set (Saunders et al., 2012; Garnica et al., 2014; Nemri et al., 2014). Upadhyaya et al. (2015) annotated a total of 22,391 expressed gene sequences in the pan-genome assembly based on RNAseq transcripts from isolated haustoria and germinated spores. About 20% of the genes showed differential expression between haustoria and germinated spores, reflecting different biological processes in these cell types. A total of 520 effector candidates were predicted based on the criteria of encoding secreted proteins without additional membrane spanning domains (1924 in total in the genome) and showing either greater than two-fold upregulation or high expression (>100 RPKM) in haustoria. Similarly, genomic and transcriptomic studies on other rust fungi have revealed large sets (500–1500) of potential effector genes (Cantu et al., 2011, 2013; Duplessis et al., 2011; Hacquard et al., 2012; Garnica et al., 2013; Link et al., 2014; Nemri et al., 2014).

KEY CONCEPT 5 **Effector candidates**

The *Pgt* genome contains over 500 genes predicted to encode secreted proteins that are preferentially expressed in haustoria. These genes comprise a set of most likely *Avr* gene candidates.

The large numbers of putative effectors in rust genomes make it difficult to prioritize candidates for functional studies. To address this challenge, other unbiased effector prediction approaches have been developed. Saunders et al. (2012) used a method to rank predicted gene families (“tribes”) according to their likelihood of containing effectors based on a combination of properties associated with known effectors such as a predicted secretion signal, small size, high number of cysteines, *in planta* induction, and their genomic location. Whilst this method is powerful for finding expanded effector gene families in rust fungi, it does not consider effector genes that are not part of such families. Sperschneider et al. (2014) used comparative genomics to classify proteins into those that are generally conserved across fungi and those that are specifically associated with pathogenic fungi, including those that are specific to rust fungi. This analysis revealed that *in planta* up-regulated genes that are under diversifying selection are found almost exclusively in pathogen-associated gene families. In particular, a small set of 42 high-confidence effectors was identified that are rapidly evolving within the *Puccinia* genus, consistent with co-evolution with host *R* genes or virulence targets. While adaptive evolution analysis is a powerful approach for prioritizing effector candidates, it can only be applied to genes for which distantly diverged sequences or population genomics data are available. More recently, a machine learning approach has been developed to make effector predictions in fungi without making assumptions on their protein properties, and can complement other approaches to identify novel rust effectors (Sperschneider et al., 2015).

## Genetic and functional tools: Associating genotype and phenotype

Genome sequencing and effector predictions have provided a critical resource for *Pgt* virulence studies, but the determination of *Avr* gene function will depend on genetic and functional evidence to associate these genes with avirulence/virulence phenotypes **identifying Avr genes**. In the flax rust system, a mapping family segregating for multiple *Avr* loci was essential for identification of *Avr* genes (Dodds et al., 2004; Catanzariti et al., 2006). A similar resource is available in *Pgt*, with an F_2_ family population of 81 individuals developed from a cross between two North American strains, including CDL 75-36-700-3, (Zambino et al., 2000). This family segregates for avirulence/virulence on 10 *Sr* genes, eight of which segregate as a single dominant genes. Genome-wide mapping of SNPs is currently being undertaken to anchor these onto genome scaffolds and thereby identify linked effector candidates, and additional segregating populations are also being generated.

KEY CONCEPT 6 **Identifying Avr genes**

Genetic variation for virulence in segregating populations, clonal lineages and derived mutants allows association of sequence variation in gene candidates with avirulence phenotypes.

The Australian *Pgt* collection also provides an excellent genetic resource for *Avr* gene identification. This asexual pathogen population falls into clonal lineages that have largely evolved by sequential mutation to overcome specific *Sr* genes. The extensive pathotype analysis from the University of Sydney annual pathogen surveys provides the necessary phenotypic data to correlate gene sequences with specific virulence changes. Upadhyaya et al. (2015) initiated an analysis of this material focusing on two field isolates within the race 21 lineage that had acquired virulence on four additional resistance genes (*Sr5, Sr11, Sr27, SrSatu*). Comparison of these genome sequences with the original 21-0 progenitor pathotype identified a total of 25 effector candidates containing sequence changes that could be responsible for conferring these virulence phenotypes.

Both of these resources are however limited by the genetic variation that occurs within a single segregating family or collection of field isolates, which mostly represents *Sr* genes that have been used historically and subsequently overcome. Identifying *Avr* genes corresponding to important new *Sr* genes will require a different approach. A promising strategy at this time is to use a mutagenesis approach to generate genetic diversity that can then be associated with sequence and virulence changes. The feasibility of this approach was demonstrated in early mutagenesis experiments (Luig, 1978). Because *Pgt* is dikaryotic, the genotype of the parental race (homozygous or heterozygous) will affect the ease of identifying virulence mutations. Indeed, spontaneous virulence mutants provided a valuable resource in cloning *Avr* genes from *M. lini*, but were only detected for heterozygous *Avr* loci (Dodds et al., 2004; Catanzariti et al., 2006). Thus the use of genetically diverse *Pgt* isolates in mutagenesis experiments is advantageous because the mutability of different races is likely to differ on specific *Sr* genes. Recently, a spontaneous mutant of *Pgt* virulent to the broadly effective and now cloned *Sr50* was identified (Mago et al., 2015). Sequencing experiments are underway to determine the nature of the genetic alteration in this race. We are also conducting new screens for both spontaneous and EMS-induced virulent *Pgt* mutants against a range of *Sr* genes to identify new *Avr* gene candidates.

Lastly, confirmation of the identity of genetically defined *Avr* genes will require demonstration of activity through functional assays. In many disease systems, this can be achieved by either transformation of a virulent pathogen to test for an avirulent phenotype, or by transient expression of the candidate effector in a resistant host to test for defense induction. Although transformation of flax rust is possible, it relies on silencing a previously identified *Avr* gene for selection (Lawrence et al., 2010) and no transformation system has yet been developed for *Pgt* or other rust fungi. Likewise Agrobacterium-mediated transient protein expression is not available for wheat. Other potential assays include bacterial systems developed in *Pseudomonas* and *Xanthomonas* species that can deliver effector candidates via Type 3 Secretion (Sohn et al., 2007; Thomas et al., 2009). Recently wheat compatible versions of these delivery systems have become available (Yin and Hulbert, 2011; Upadhyaya et al., 2014). Other possibilities include generating transgenic plants expressing *Avr* candidates and crossing these to the *Sr* resistant line to test for cell death defense induction and transient expression by biolistic transformation (Jones et al., 1994; Leister et al., 1996; Jia et al., 2000). Host induced gene silencing of candidate genes may also provide a useful functional assay (Panwar et al., 2013; Yin et al., 2015). Where specific *Sr* genes have been cloned, these may be co-expressed with the *Avr* candidates in a heterologous system, such as *Nicotiana benthamiana*. This approach was used recently to confirm the identity of *AvrPm3f* from wheat powdery mildew as it triggered cell death when recognized by the corresponding wheat *Pm3f* resistance gene (Bourras et al., 2015).

## Future perspectives

Keeping stem rust at bay has not been an easy task; however, for over 80 years breeding for disease resistance and the deployment of resistant cultivars has been a powerful strategy to lessen the frequency of stem rust disease outbreaks (Leonard and Szabo, 2005). Nonetheless, stem rust continues to threaten grain production and is a serious problem in regions of the world where small-scale farming plays a pivotal role in agriculture and economic resources are limited. One lesson we have learned is that the durability of genetic resistance depends on the manner in which it is deployed. Employing multiple resistance genes is essential to reduce the likelihood of new virulent variants emerging. This strategy will require careful selection of *Sr* genes to be deployed in new wheat cultivars, informed by knowledge of the corresponding *Avr* genes and their population genetics. In light of this, the identification of effectors in *Pgt* and understanding the underlying mechanisms that generate genetic variability in *Pgt* are two research priorities. The coordinated international surveillance efforts led by the Borlaug Global Rust Initiative and the Durable Rust Resistance in Wheat Project during the last 8 years have created a platform to enable pathogenomics studies in recently emerged virulent isolates from multiple points of origin. In combination with historical collections of *Pgt* isolates these resources provide a valuable experimental system to address these research priorities. Recent breakthroughs in cloning *Sr* genes from wheat have provided tools such as perfect molecular markers to facilitate the production of gene pyramids or stacks. In parallel, these advances also provide materials for generating transgenic events with multiple *Sr* genes at a single location which will greatly simplify the breeding constraints of multiple unlinked genes. Cloned *Avr* genes will provide a means to verify the function of individual *Sr* genes in such combinations. The ability to rotate between stacks with different R gene components would also contribute to resistance durability (McDonald, 2014). Finding new stem rust resistance genes means continuing laborious germplasm screens in crop varieties or related species (Rouse M. et al., 2011; Rouse M. N. et al., 2011). Additionally, non-host systems such as the grass *Brachypodium distachyon* may provide novel sources of resistance genes from outside the wheat and barley gene pool (Ayliffe et al., 2013; Figueroa et al., 2013, 2015). Overall, the existing genetic and genomic resources available to study *Pgt* (Zambino et al., 2000; Duplessis et al., 2011; Upadhyaya et al., 2015), wheat (Mayer et al., 2014), barley (The International Barley Genome Consortium, 2012), and *B. distachyon* (The International Brachypodium Initiative, 2010; Gordon et al., 2013) hold significant promise to strengthen genetic resistance approaches to battle stem rust.

## Author contributions

All authors listed, have made substantial, direct and intellectual contribution to the work, and approved it for publication.

### Conflict of interest statement

The authors declare that the research was conducted in the absence of any commercial or financial relationships that could be construed as a potential conflict of interest.
